# Supplementary material for: Cyano-B12 or Whey Powder with Endogenous Hydroxo-B12 for Supplementation in B12 Deficient Lactovegetarians
Source: Nutrients. 2019 Oct 6;11(10):2382. doi: 10.3390/nu11102382 (PMC6835307; doi:10.3390/nu11102382)
Supplement: Supplementary file 1 [file nutrients-11-02382-s001.pdf]

Supplementary material for:

Cyano-B12 or whey powder with endogenous hydroxo-B12 for supplementation in B12 deficient lactovegetarians

Sadanand Naik [1*], Namita Mahalle [1], Eva Greibe [2], Marie S. Ostenfeld [3], Christian W. Heegaard [4], Ebba Nexo [2] and Sergey N. Fedosov [4*]

1 Department of Pathology, Deenanath Mangeshkar Hospital and Research Center, Pune, India; pnmahalle@gmail.com (N.M.)

2 Department of Clinical Biochemistry and Institute of Clinical Medicine, Aarhus University Hospital, DK-8200 Aarhus N, Denmark; evagreibe@gmail.com (E.G.); ebbanexo@rm.dk (E.N.)

3 Arla Foods Ingredients Group P/S, DK-8260 Viby, Denmark; marie.stampe.ostenfeld@arlafoods.com (M.S.O)

4 Department of Molecular Biology and Genetics, Aarhus University, DK-8000 Aarhus C, Denmark; cwh@mbg.au.dk (C.W.H)

* Correspondence: sadu_naik@rediffmail.com (S.N.); snf@mbg.au.dk (S.N.F.); Tel: +91 20 66023194 (S.N.); +45 87155521 (S.N.F.)

Supplementary Table S1. Nutritional composition of whey powder (Lacprodan® DI-8290) as per manufacturer's[a] product specifications.

Composition	Amount (dry basis)
Protein %	81,5
Lactose %	2
Fat %	8
Ash %	2.2

[a]*Lacprodan® DI-8290 was procured by Arla Foods Ingredients Group P/S, Viby, Denmark.*

Supplementary Table S2. Fitting parameters of the markers, responding to the treatment with CN–B12 capsules, or whey powder.

**Treatment and P_i number**	**CN–B12 capsules fitting P_i (*p* of reference value)**		**whey powder fitting P_i (*p* of reference value)**	
ΔB12	Equation 1			
$P_1 \pm SE_1$	0.0 ± 18	(1.0)	0.0 ± 4.1	1.0
$P_2 \pm SE_2$	97 ± 20	$(7 \cdot 10^{-6})$	47 ± 13	0.0003
$P_3 \pm SE_3$	0.69 ± 0.42	(0.1)	0.29 ± 0.21	0.17
(*p* overall)		**$(7 \cdot 10^{-8})$**		**$(5 \cdot 10^{-5})$**
ΔholoTC	Equation 2			
$P_1 \pm SE_1$	0.0 ± 5.0	(1.0)	0.0 ± 2.8	(1.0)
$P_2 \pm SE_2$	60 ± 10	$(9 \cdot 10^{-8})$	38 ± 6	$(3 \cdot 10^{-9})$
$P_3 \pm SE_3$	8.0 ± 0.9	$(2 \cdot 10^{-13})$	8.2 ± 0.9	$(8 \cdot 10^{-16})$
(*p* overall)		**$(2 \cdot 10^{-20})$**		**$(3 \cdot 10^{-24})$**
R(Hcy)	Equation 2			
$P_1 \pm SE_1$	1.00 ± 0.04	(1.0)	1.00 ± 0.02	(1.0)
$P_2 \pm SE_2$	–0.38 ± 0.09	$(4 \cdot 10^{-5})$	–0.47 ± 0.07	$(6 \cdot 10^{-9})$
$P_3 \pm SE_3$	9.4 ± 1.6	$(9 \cdot 10^{-8})$	6.8 ± 0.7	$(1 \cdot 10^{-17})$
(*p* overall)		**$(4 \cdot 10^{-12})$**		**$(6 \cdot 10^{-26})$**
R(MMA)	Equation 2			
$P_1 \pm SE_1$	1.00 ± 0.06	(1.0)	1.00 ± 0.05	(1.0)
$P_2 \pm SE_2$	–0.67 ± 0.12	$(1 \cdot 10^{-7})$	–0.34 ± 0.07	$(4 \cdot 10^{-6})$
$P_3 \pm SE_3$	8.2 ± 1.0	$(4 \cdot 10^{-13})$	6.4 ± 1.1	$(1 \cdot 10^{-7})$
(*p* overall)		**$(5 \cdot 10^{-20})$**		**$(4 \cdot 10^{-13})$**

The bold figures for the overall probabilities highlight the high levels of significance for deviation from a "zero response".
